# Perceived risk and condomless sex practice with commercial and non-commercial sexual partners of male migrant sex workers in London, UK

**DOI:** 10.12688/f1000research.73248.1

**Published:** 2021-10-11

**Authors:** Elisa Ruiz-Burga

**Affiliations:** 1 University College London - Great Ormond Street Institute of Child Health, London, WC1N 1EH, UK

**Keywords:** HIV, STI, male migrants, sex work, condomless sex

## Abstract

**Background:** Since the emergence of HIV and the AIDS pandemic, the majority of risk-reduction interventions have been centred on the use of condoms in sex workers.

**Methods:** This qualitative study recruited 25 male migrant sex workers in London to understand their risk perception and condomless sex experiences within the context of sex work and private life. The data was collected using face-to-face interviews, analysed using thematic analysis, and the findings interpreted through the theory of planned behaviour.

**Results: **The themes explain that condomless sex with clients occurred when participants consciously accepted to perform this service deploying a risk assessment of clients, faulty strategies, and sexual practices to reduce their risk; or when they lost control because of recreational drugs, feeling attraction to clients, were in precarious circumstances, or were victims of violence. Conversely, condomless sex with non-commercial partners occurred according to the type of relationship, with formal partners it was rationalised through emotional aspects attached to this kind of relationship, while with casual partners it was connected to sexual arousal and the use of alcohol and drugs.

**Conclusions:** Reinforce educational interventions to deliver STI-HIV information, enhance the use of condoms, and to address specific contextual factors that facilitate condomless practice with commercial and non-commercial sexual partners.

## Introduction

Since the emergence of HIV and the AIDS pandemic, sex workers were considered a highly vulnerable group ^1^ because of their high-risk exposure to acquire these infections compared to adult non-sex workers. ^2^ Across the globe, approximately 8% of the newly HIV cases are reported among sex workers. ^3^ Of serious concern are male sex workers (MSW) who are at more HIV risk than female sex workers (FSW). Receptive anal intercourse and insertive anal intercourse are considerably at higher risk of HIV transmission than vaginal sex. ^4^ Further, MSW are greatly affected by other sexually transmitted infections (STIs). ^5^^–^^7^ In response to AIDS and the concurrent increase in HIV prevention research, numerous interventions that aimed at decreasing the risk of infection have been conducted to reduce the practice of condomless sex. ^8^^–^^11^ After decades, there is still a debate about the success of these interventions - while some authors claim that new infections are yet associated to an inconsistent use of condoms, ^12^^,^^13^ others argue that MSW are using them more regularly, for either insertive anal sex or receptive anal sex. ^14^^–^^16^

A large number of sex workers in Europe are migrants who are living and working in disadvantaged circumstances, facing isolation and social exclusion. ^17^^–^^19^ Moreover, migrant sex workers are extremely exposed to HIV and STIs due to their overlapping risks ^3^^,^^6^^,^^20^ and structural inequalities that can create difficulties to use health services for HIV prevention, testing and treatment ^17^^,^^21^^,^^22^ in some European countries. For example, it has been reported that male street sex workers, in particular illegal migrants in Germany, have lack of access to health care services due to their socio-legal position. ^23^ This is a significant aspect as non-European sex workers who are highly mobile in Europe ^19^ and are under different migration status, ^24^ can be impacted by the legislation and internal policies of each country that determines their access to the health care services. ^17^ In the United Kingdom, an important proportion of the sex work population is represented by male migrant sex workers (MMSW) who mostly work indoors in London. ^25^^–^^27^ Reports show that they are mainly from Europe, Latin America and the Caribbean countries. ^26^^,^^28^ Epidemiological and qualitative research have demonstrated that these migrants utilize national health care services (NHS), including sexual health clinics. In this manner, they can be tested for HIV and other STIs, receive counselling, adequate information, and a provision of condoms and lubricants. ^24^^,^^29^ However, a study using national data demonstrated that although the use of sexual health clinics does not vary between British MSW and MMSW, the latter group seem to be more exposed to HIV and chlamydia infections. ^29^

This paper explores the risk perception and condomless sex experiences of MMSW with commercial and non-commercial sexual partners, as discrepancies have been reported in the use of condom according to the type of sexual partner, ^14^^,^^16^^,^^30^^–^^32^ and the sexual role performed during the act. ^27^ In this manner, this paper aims to contribute to lessons learnt and recommendations for future educational interventions for this highly vulnerable group. This paper is pertinent in an era when the role of behavioral interventions is evolving with the advent of efficient alternatives of prevention such as pre-exposure prophylaxis (PrEP) and post-exposure prophylaxis (PEP). Despite of the efficacy demonstrated by the PrEP scheme, it requires high adherence to the treatment, strict laboratory monitoring, ^33^ and more importantly, the use of supplementary methods of prevention such as the use of condoms to further reduce the HIV risk, and specifically for protection against other STI; since PrEP only protects against HIV. ^34^

## Methods

### Study design and recruitment

This qualitative research was carried outed between May 2013 to August 2015. Convenience sampling method was used to recruit men aged 18 and over, who were non-British, lived in the country for at least one year, and who had worked or were still working as sex workers. The main recruitment sites were sexual health clinics and projects that provide health and social services to sex workers in London. Health professionals and health workers took part in the recruitment by providing the participant information sheet (PIS) and flyers to potential participants.

### Data collection and analysis

An interview guide was prepared using pertinent literature and first-hand information obtained from researchers and health professionals working in projects or programmes focused on sex work in London. The questionnaire ( Underlying data) ^35^ was tested on three MMSW, however the results were not included in the final analysis. Participants were interviewed in counselling rooms and small meeting rooms located in St. Marys hospital and City, University of London to secure their privacy. Literal transcription of the recordings was printed and revised by each participant to confirm their accuracy. In this manner, the risk of misinterpretation and misunderstanding was minimised. The software ATLAS.ti version 8·0 was used to store the data and thematic analysis was performed as previously described. ^36^ Coding rules were established as well as a clear definition of the themes to avoid ambiguity or inconsistency. For this investigation, 'condomless sex' was defined as any penetrative or receptive sexual intercourse (oral, vaginal, or anal) without using condoms. Conversely, 'safer sex' was specified as the use of condoms for the aforementioned practices. The emergent themes were interpreted using the theory of planned behavior (TPB) ^37^ framework. This theory is based on the significance of attitudes, norms, and perceived control to explicate different forms of risky behaviours, ^37^ and to plan health interventions. ^38^ In this manner, the analysis was focused on 1) attitudes towards the use of condoms: a result of personal beliefs about condoms; 2) subjective norms derived from participants’ perception of what others think about condoms (normative beliefs) and their motivation to comply with norms, and 3) perceived behavior control: participant’s beliefs about the degree of control they have over the use of condoms during the intercourse.

### Ethical considerations and consent

This study was revised and approved by the Ethics Committee of City, University of London (18 April 2013, Ref: PhD/12-13/18), by the NRES London Central Committee (9 October 2013, Ref:13/LO/1306, IRAS ID: 132947), and by the Research Committee of St. Mary’s Hospital (15 January 2014, Ref:13SM1864). Written informed consent was obtained from all individual participants included in the study. This form was revised and approved by the ethics committee.

## Results

### Disclaimer

Due to the explicit nature of the interviews some quotes have been edited for clarity.

### Main characteristics of the participants

In this study a total of 25 MMSW (n = 25) participated. This sample was almost evenly represented by men from Latin-America and Europe. The socio-demographic characteristics of participants as well as their patterns of migration, and entrance into sex work suggest their diversity ( Table 1). The whole group was operating as independent internet-based escorts, providing sexual services to men and women. The latter in the context of sexual services for ‘couples’ (man and woman). The analysis shows two dominant themes and distinct subthemes:

**Table 1.** The socio-demographic, immigration status and sexual health characteristics of participants.

Home-country	Total (n = 25)
Brazil	10 (40%)
Colombia	02 (8%)
Bulgaria	02 (8%)
Spain	06 (24%)
Italy	02 (8%)
Portugal	01 (4%)
Latvia	01 (4%)
Nigeria	01 (4%)
**Age** (mean [range], years)	33 (24-44)
**Patterns of immigration to the UK**	
Direct migration	14 (56 %)
Multi-stage migration	11 (44%)
Age at emigration (mean [range], years)	24 (13-40)
Years living in the UK (median [range], years)	06 (1-23)
**Entrance into sex work**	
Age (median [range], years)	27 (14-41)
Years in sex work (mean [range], years)	06 (1-16)
**Level of education achieved**	
Primary	02 (8%)
Secondary	10 (40%)
Further/higher education	13 (52%)
**Sexual orientation reported**	
Gay	19 (76%)
Bisexual	06 (24%)
**Currently using recreational drugs at work**	
Yes	15 (60%)
No	10 (40%)
**Currently using recreational drugs in personal life**	
Yes	18 (72%)
No	07 (28%)
**STI reported**	22/25 (88%)
Syphilis	12 (48%)
Gonorrhea	15 (60%)
Chlamydia	16 (64%)
Herpes	02 (8%)
Genital warts	03 (12%)
HIV	03 (12%)

**A. Condomless sex with commercial sexual partners**

This theme explains the perspective of the use of condoms within the context of sex work and experiences of condomless sex that contains three sub-themes:

1. Policy on condom use with clients

It describes the attitude and perceived norm about the use of condoms with clients. The statements indicate that the entire group of participants were aware of the HIV and other STIs, claimed to be risk averse, and more importantly, stated a consistent use of condoms as a perceived norm because of their work. In line with this, many of them have made explicit their rejection to condomless sex in their online adverts:

*“Always, always condom, nothing without a condom, never, ever, ever. They can pay me any money, there are some offers, and some people asked me - do you do [condomless sex]? For that reason, in my profile I wrote no [condomless sex], don’t even ask me”*

Consistently, the majority of participants expressed a favourable attitude towards safer sex. For some, using condoms with clients was a way to differentiate sexual services from having sex in their personal life. Others thought that condoms were useful devices to avoid poor hygiene, odour, and some bodily features of clients that they find unattractive or dislike (e.g., overweight, excessive corporal hair). Most of the participants said that they especially demand the use of condoms to provide services such as vaginal intercourse, ‘full-service’ for men (usually include anal sex) and performing “passive” sexual role service (receptive anal sex):

*“I offer a full service, a complete service but I try to be specific because people always ask you if you do without condom, So I always say condom, I won’t give you my phone, I am a very discrete person and I only do outcalls.”*

By contrast, other participants admitted an unfavourable attitude towards condoms. While the most mentioned concerns of losing clients that reject condoms, few did not like their use as it reduced their sexual arousal, especially with erection which caused difficulties in their sexual performance:

*“I know that I won’t have many clients if I insist on having oral sex with a condom, so I prepare to take that risk that is the only one that I prepare to take the chances.”*

*“I don’t enjoy at all when I use a condom for [oral sex] because it is like you are sucking a rubber and I just get soft* [lose the erection] *when I wear a condom for a [oral sex], once again it is because of it something like squeeze it is a bit weird.”*

The discrepancy between these two different perspectives suggests that risk awareness and intention of using condoms do not guarantee safer sex.

In addition, the analysis of the narratives showed that condomless sex with clients occurred in two different scenarios. In the first scenario participants perceived control of the situation and made a risk-taking decision to dismiss the use of condoms, and in the second, they lost control of the situation that ended in a condomless sex event. These are described under the following sub-themes:

2. Decision-making process to accept condomless sex

This sub-theme explains the decision-making process that participants applied to dismiss the use of condoms with clients. They used two main processes a risk assessment of the clients, and performing sexual practices that participants catalogued as *'low-risk'* in contracting HIV and other STIs. The participants assessed the risks of the client based on their physical appearance, followed by a subtle physical examination to identify sores, warts or blisters on genitals or rectum, or presence of penile discharge. Through this practice participants accepted condomless sex with customers that were labelled as *‘healthy’* and *‘clean’* (e.g., absence of warts):

*“I [had sex with] him all without condom, but I was not a risk as I could tell that I was probably the second or third person who he has sex with. I think I could tell about everything I do not think he has anything because he probably has very little sexual life.”*

The participants also considered some social and demographic characteristics to rank clients as *'low risk'* or *'high risk'.* In this instance, participants favoured clients who were *'married men'* for condomless sex as they were perceived as *‘straight’* men who *‘only have sex with their wives.’* In the same way, participants considered their *‘regular’* clients with whom they had established a long-term and trustworthy relationship, as ‘low risk’:

*“For example, yesterday I had a man from Barbados who looked very healthy, but I know he is from a high-risk region for these diseases. The guy was very clean, he was very nice”*

*“There are a couple of people that I don’t use a condom because I know them for quite long time. I know it is not a good policy. I know I should use a condom with everybody and that’s it, but there are few people that I do that.”*

A second procedure to accept condomless sex with clients was the selection of sexual practices that participants considered as *'low-risk.'* By far, condomless oral intercourse (COI) was the most frequent practice. Some participants mentioned that as additional strategies of protection they reduce the time for COI and avoid contact with the client’s semen. Although, condomless insertive oral intercourse (CIOI) and condomless receptive oral intercourse (CROI) were equally reported by participants, some assigned different levels of risk to each:

*“I know it is less risky when I suck him than when he sucks me or to kiss him. But it is not for everybody, depend on of the situation”.*

Another recurrent risk reduction practice mentioned was performing condomless anal sex as the active sexual role (penetrative anal intercourse) instead of a passive role (insertive anal intercourse), as it was perceived as less risky:

*“Part of me think I am mainly top, I normally do not get people to [have sex with] me, I [have sex with] them, I am mainly top, and that is a very low risk,* [censored] *! I am not at risk because it is the very little rate to catch something if I am mainly top.”*

*“I think, I am in this scene, I am earning money, but I am also scared because this is very risky, but I always pray to God please nothing bad happen.”*

3. Structural factors determining unplanned condomless anal sex

This sub-theme describes the role of four main factors that made participants to *'lose control'* or to be under pressure to perform condomless anal intercourse. One of the most recurrent conditions was the use of recreational drugs that provoked a strong sexual arousal among participants. Many of these events occurred when they were providing *‘overnight' or 'chemsex’* services:

*“With crystal meth your brain is still more there, but with mephedrone you do not even think straight so much, you are so* [aroused] *that you do not even think, you only want sex, if I take mephedrone I know I am not going to use condoms”*

A second recurrent and independent condition was feeling strong attraction to a client:

*“Actually, I wasn’t on drugs, this time I wasn’t on drugs, I came to see this guy in the Ritz Hotel, and he was an Arabic, he was about my age, and he was so gorgeous! Sexy, he was like my God! I just wanted to eat him alive, he was so sexy and then, you know what actually I did it without condom”*

A third factor driving condomless anal sex was the financial reward offered by clients, which was mostly reported by participants who were in precarious conditions. In these situations, the participants felt that they could not reject the offer:

*“I had a client once, the same client three times because that client, he pays very well, much more that what I asked”.*

*“I am at risk if you ask me how I feel about it, not very safe, not very clever. But I gamble for the best, I need the cash, I need the cash for food, I need the cash for transport, and I need to get out of this hole because I smile when I meet new people and everything, but when I am alone is not easy.”*

*“Once I was really bad about money and a client called me and he wanted to take drugs also if you don't take drugs, you can last all that you need or you can even cope with the client.”*

The fourth condition describes scenarios in which participants were overpowered by clients who removed or broke condoms or took advantage of the dynamic during the sex session to perform condomless anal intercourse. This condition was usually reported by the participants who offer a ‘passive’ sexual role as part of their sexual services:

*“I was having sex with a guy who was doing as active, and you know suddenly I saw him with the condom on his hand and I asking him, ‘Were you [having sex with] me without a condom?!”*

In few cases, participants reported that these events occurred in a context of physical and verbal violence perpetrated by clients, or within a context of drugs use:

*“We were taking cocaine and drinking, I drank so much that day and I passed out […] few hours later, I woke up and the reason that I woke up was because something was painful, ok? And the painful thing was that he was [having sex with] me on my sleep, he was [having sex with] me on my sleep and without condom.”*

**B. Condomless sex with non-commercial sexual partners**

This theme describes experiences of unpaid or non-commercial condomless sex, which was defined as sexual acts without the use of condoms that were performed without any intention of material or economic reward. In general, many participants declared a more inconsistent use of condom with non-commercial sexual partners than with clients:

*“Then it happened again, but he wasn’t a client he was a person that I met, a casual partner and again it was three months of waiting for the test and I was - Oh my God, I shouldn’t do it again!”*

*“I haven’t been in risk. My sexual practices are very low risk in the context of work, and the only person with I have been in high risk is with my ex-partner. During the time when we knew that he got infected we used protection until he completed the treatment”*

This theme contains two sub-themes to differentiate condomless sex practice with formal sexual partners from casual sexual partners. Most of the participants reported having casual partners along with a formal partner in the past year.

The sub-themes are described below:

1. Condomless anal sex with formal partner

The category of formal sexual partner was used by participants to describe people with whom they had a romantic, stable/regular or committed relationship. Almost half of the group reported to have male formal sexual partners. Some mentioned that these partners were also working as escorts, even few worked together. Most importantly, majority reported an irregular or complete lack of condom use with these partners. They decided not to use condoms when their partners agreed to just have sex with them, and/or knew both were HIV negative:

*“When I am dating someone if we both checked [got tested for HIV] and we both are fine, we do not use condom, like my ex that we split up two months ago, we were together for a year as we never use condoms, but I knew he only was sleeping with me”*

For these participants condomless anal sex represented pleasure, intimacy, and commitment with their partners:

*“Have sex without condom with my ex-partner wasn’t good idea, even if that gives me more pleasure and it gives me more intimacy because sex between us hasn’t been the most important part of our relationship”*

Coherently, few participants said that they *'always'* used condoms with their formal partners because they knew that one of them was HIV positive (serodiscordant couples):

*“He found out that he was HIV positive and then at that time I got syphilis from him and at that time I wasn’t working as an escort I was working as a cleaner and I didn’t get the HIV, so I got treated for syphilis, he got treated as well and from then we started to have sex with condom.”*

2. Condomless anal sex with casual partners

Casual sexual partner was defined as people with whom participants engaged in sexual intercourse without a sense of commitment or emotional attachment. They mainly met casual partners using dating mobile applications, websites or in places such as clubs, saunas, or clubs. These participants decided not to use condom with these partners to satisfy their pleasure and personal enjoyment. Some admitted that they perceived the use of condom was optional:

“ *We are humans and sex is the most animal part of us, you know, we are animals completely, so you cannot always control it, you have to accept it, if you always are having sex […] that is why you do without condom and see what happen.”*

*“If someone carry a condom, then we do it with condom, or we just leave the condom around and try to see how it goes.”*

However, it is important to mention that many participants also acknowledged the role of recreational drugs and alcohol consumption as well as feeling sexually attracted to casual sexual partners in the practice of condomless anal intercourse:

*“When you are in drugs the only thing that you want is to have sex, well it depends, in my case I only wanted to have sex, for free, sex with people that if I could be rational, I wouldn’t like to have sex with, and exposing yourself to catch anything.”*

*“The very last time was about 6 months ago and that was with a neighbour, a very, very hot Spanish guy who came around and we had some fun and when he started [having sex with] me without condom”*

## Discussion

This study has used the theory of planned behaviour to explore the risk perception and condomless sex experiences of 25 male migrant sex workers with commercial and non-commercial sexual partners. Unlike other research, ^39^ the participants of this study knew the risks of HIV and other STIs, self-reported risk adverse, and consistently declared their position against condomless sex in their online adverts. However, despite their perception of safer sex as a norm and their intention of using condoms with clients, they revealed that condomless sex was a frequent practice. ^40^^–^^42^ In accordance with this, the participants exposed an unfavourable attitude towards condoms due to displeasure, concerns of losing clients that reject condoms, and issues affecting their sexual response and performance. The latter, not so often acknowledged, highlights the importance of male sexual arousal in this type of work. ^43^ In addressing past events of condomless sex with clients, this study identified a perceived behavioural control among participants that made this high-risk decision based on signs of physical evidence for HIV and other STIs in clients, assessing client’s social and sexual risk characteristics, and performing sexual practices that they considered 'low risk', to lessen the risk of transmission. These practices, that indicate a self-protective behaviour, demonstrate the persistence of inaccurate information about HIV and other STIs. ^15^^,^^44^^–^^47^ In addition, this study identified factors that made the participants lose their perceived control and drove them to condomless anal sex. One of main factors was the use of recreational drugs with clients. ^48^^–^^50^ Authors describe the use of drugs with clients as a social aspect of their occupation, ^51^ while improving their performance. ^52^ Further, some argue that this is a difficult aspect to avoid with clients who are regular drug users. ^49^ Another interlinked factor was feeling sexually attracted to clients that implied opting for personal pleasure over a professional perspective. ^53^^,^^54^ A third factor was the precarious situation that made participants to accept the financial reward offered by clients. ^55^^–^^58^ Similar to other studies, ^55^^,^^58^ physical domination and verbal violence perpetrated by clients was the other aspects facilitating condomless experience, which validates MSW's vulnerability regardless of the situation. ^59^

Within the context of private life, this study found that differences in the perspective of condomless sex was related to the type of non-commercial sexual partner. As such, most of participants dismissed the use of condoms with formal partners as they were emotionally attached to these relationships. Participants also said that they agreed not to use condoms when they both were HIV negative and keep this practice strictly among themselves. Although condomless sex with formal partners was perceived as a safer practice, some participants had episodes of STIs that were associated to these sexual partners. Besides, it is worth noting that in line with other studies ^43^^,^^60^ few participants reported a consistent use of condoms with formal partners when they were HIV-serodiscordant. Regarding condomless sex practice with casual sexual partners, participants said that it was more frequent with these sexual partners than with their clients. They connected this practice to strong sexual arousal and the use of drugs and alcohol. Drug use mainly initiates sexual interaction between gay and bisexuals, ^40^^,^^61^ and more importantly, ^62^^,^^63^ it facilitates sexual acts. ^64^^,^^65^ Of serious concern is that these substances affect the perception and response to risk, directing the behaviour to high-risk sexual practices, ^66^ and consequently, increase the possibility of contracting HIV and other STIs. ^67^^,^^68^ This finding suggests the use of recreational drugs and attraction to the client are significant factors that intersected both private and sex work experiences of the study participants, and reinforce claims that condomless sex with casual partners can be a predictor of condomless sex with clients. ^53^ Additionally, these results support the perspective that the type of sexual partner chosen in the MSW’s personal life can also be a risk factor. ^40^^,^^61^

Overall, participants that experienced condomless sex visited sexual health clinics to have screening tests for HIV and other STIs, as they felt exposed to contracting these infections. The most concern and anxiety was expressed for HIV, therefore almost all participants had requested for post-exposure prophylaxis (PEP) to reduce their chances of contracting this infection. ^69^^,^^70^ Some had received PEP more than twice in the past 12 months. Few admitted to not continuing with their PEP treatment due to the adverse effects. These findings raise concerns of possible seroconversions when considering the poor medication adherence. ^71^ It is relevant to mention that nearly the whole group of participants (22/25) had been diagnosed with one or more STIs including HIV ( Table 1).

### Strengths and limitations

Interpretation of the findings and the evaluation of their significance should be made considering the limitations of this study. For instance, the study design prevents the generalization of the findings. Also, limiting the recruitment of participants to sexual health clinics and health projects in London due to the recommendation of the research ethics committees, restrict the results only to the perspective and experiences of migrants who attend these services. Even with these limitations, this study is one of the few on male migrant sex workers in the UK that captures their experiences of high-risk sexual behaviour in detail. In addition, the heterogeneity of the sample provided a rich qualitative data on MMSW’s risky sexual behaviour with commercial and non-commercial sexual partners. Furthermore, this study provides in-depth socio-behavioural insights for designing more effective and tailored interventions for MMSW self-identified as homosexuals and bisexuals.

## Conclusions

Participants experienced condomless sex with commercial sexual partners as risk-taking decision that intuitively triggered a set of risk reduction practices, which may not work effectively as they were based upon misinformation. Condomless sex with clients also occurred in a context of perceived loss of control with recreational drug use, experiencing precarious conditions, physical domination and verbal violence perpetrated by clients. These findings challenge claims that recreational drugs are not problematic among male escorts, ^72^^–^^74^ that they work in safer environments, obtain higher earnings, and can control work conditions. ^73^^,^^75^^,^^76^ In addition, condomless sex with non-commercial sexual partners was also a common practice, but clearly differentiated by the meanings attached to formal and casual partners. More importantly, this study found that the use of recreational drugs and attraction to the client are significant factors that intersected private and sex work experiences.

## Recommendations

The findings suggest the need to reinforce educational interventions to deliver appropriate information about the transmissibility of HIV and other STIs, improve skills of self-control, strengthen the risk-reduction counselling for those requesting PEP, and the use of condoms for those who decide to take pre-PEP as the best action to secure its success. Likewise, emphasising the relevance of training healthcare professionals to identify MMSW who use recreational drugs, and to facilitate their referral to programmes of harm reduction in substance use and mental health services. Finally, the identification of other subgroups among MMSW such as those whose partners are also sex workers, have a HIV serodiscordant partner, tend to have condomless sex with casual sex partners, and are experiencing difficult-living or working conditions, can allow for tailoring behavioural interventions.

## Data availability

### Underlying data

Repository: Perceived risk and condomless sex practice with commercial and non-commercial sexual partners of male migrant sex workers in London, UK https://figshare.com/s/cbf4a21a9d93d63472c8. ^35^

This project contains the following underlying data:

• File docx. This file contains the blank interview questionnaire.

Data are available under the terms of the Creative Commons Zero “No rights reserved” data waiver (CC0 4.0 Public domain dedication).
