# Perceived risk and condomless sex practice with commercial and non-commercial sexual partners of male migrant sex workers in London, UK

**DOI:** 10.12688/f1000research.73248.2

**Published:** 2023-10-19

**Authors:** Elisa Ruiz-Burga

**Affiliations:** 1 University College London - Great Ormond Street Institute of Child Health, London, WC1N 1EH, UK

**Keywords:** HIV, STI, male migrants, sex work, condomless sex

## Abstract

**Background:** Since the emergence of HIV and the AIDS pandemic, the majority of risk-reduction interventions have been centred on the use of condoms in sex workers.

**Methods:** This qualitative study recruited 25 male migrant sex workers in London to understand their risk perception and condomless sex experiences within the context of sex work and private life. The data was collected using face-to-face interviews, analysed using thematic analysis, and the findings interpreted through the theory of planned behaviour.

**Results: **The themes explain that condomless sex with clients occurred when participants consciously accepted to perform this service deploying a risk assessment of clients, faulty strategies, and sexual practices to reduce their risk; or when they lost control because of recreational drugs, feeling attraction to clients, were in precarious circumstances, or were victims of violence. Conversely, condomless sex with non-commercial partners occurred according to the type of relationship, with formal partners it was rationalised through emotional aspects attached to this kind of relationship, while with casual partners it was connected to sexual arousal and the use of alcohol and drugs.

**Conclusions:** Reinforce educational interventions to deliver STI-HIV information, enhance the use of condoms, and to address specific contextual factors that facilitate condomless practice with commercial and non-commercial sexual partners.

## Introduction

Since the emergence of the Human Immunodeficiency Virus (HIV) and the Acquired Immunodeficiency Syndrome (AIDS) pandemic, sex workers have been considered highly vulnerable ^1^ because of their greater risk to acquire these infections compared to adult non-sex workers. ^2^ Despite the public health efforts to respond to this pandemic, 8% of the newly HIV cases are diagnosed among sex workers across the globe. ^3^ Of serious concern are male sex workers (MSW) who are utterly affected by other sexually transmitted infections (STIs), ^4^^–^^6^ even in higher income regions as Europe where the estimated prevalence of HIV and STIs remain significantly high (12% and 48.9% respectively). ^7^ This is an important aspect taking into account that having another STI increases 2-5 times the risk of acquiring HIV in an unprotected sex event. ^8^ Thus, condomless sex, particularly during anal intercourse, still plays a critical role in HIV and STIs transmission among MSW. ^9^ After years of testing numerous behavioural interventions, ^10^^–^^13^ it is still unclear the reduction of this practice – while some authors claim that new infections are yet associated to an inconsistent use of condoms, ^14^^,^^15^ others argue that MSW are using them more regularly. ^16^^–^^18^ The introduction of the preexposure prophylaxis (PrEP) and postexposure prophylaxis (PEP) have proven to be effective, ^19^^,^^20^ but they need high adherence, strict laboratory monitoring, ^21^ and more importantly, the use of supplementary methods of prevention such as the use of condoms to further reduce the HIV risk, and specifically for protection against other STI; since PrEP and PEP only protects against HIV. ^22^ It has been estimated that an increase in condomless sex and increase of sexual partners can substantially demean the effect of the interventions. ^23^^,^^24^ Further, some studies reported lack of awareness and knowledge about these treatments among MSW, ^25^^,^^26^ whereas others reported potential side effects, ^7^ perceived risk, and practical and logistical barriers to access ^26^^,^^27^ as main barriers for their use among MSW. In this way, authors have highlighted the need to address these issues to enhance their effect on HIV prevention. ^28^^,^^29^ These findings corroborate claims that MSW still endure distinctive biological, behavioural, and structural issues that severely impact on their health outcomes. ^30^

Male sex work population is a heterogenous group across and within countries worldwide. In Europe, the proportion of MSW greatly varies from country to country as well as the type of sex work sector in which they operate. ^31^ Most of the MSW are highly mobile migrants ^32^ who live and work in disadvantaged circumstances, and face isolation and social exclusion. ^32^^–^^34^ Due to differences in their socio-legal positions in the host countries, ^35^ male migrant sex workers (MMSW) are exposed to structural inequalities such as legislation and internal policies that create barriers to access health services for HIV-STIs screening, prevention, and treatment. ^33^^,^^36^^,^^37^ For example, a study reported that male migrants working as street sex workers in Germany cannot have access to health care services. ^35^ Hence, they are extremely vulnerable to HIV and STIs due to their overlapping risks, ^3^^,^^5^^,^^38^ and the structural inequalities to access health services. ^31^ In the United Kingdom, 41% of the entire sex work population are migrants who predominantly came from Central and Eastern Europe, Latin America and the Caribbean countries, Asia and Africa. ^31^^,^^39^^,^^40^ They mainly operate in the indoor sex work sector and are concentrated in London. ^40^^–^^42^ Epidemiological and qualitative research have demonstrated that these migrants utilize national health care services (NHS), including sexual health clinics. Therefore, they can access to STI-HIV screening, receive counselling, adequate information, and a provision of condoms and lubricants. ^39^^,^^43^ However, a study using national data found that although MMSW use of sexual health clinics as much as British MSW, the former group seem to be more exposed to acquire HIV and chlamydia infections. ^43^ This brief review suggest the need of exploring the experiences of MMSW and further examining epidemiological and behavioural aspects of the condomless practice that still actively facilitate the transmission of HIV-STI. The use of condoms is still a relevant factor in the combination of HIV prevention that can enhance the effectiveness of HIV interventions.

This paper explores the risk perception and condomless practice of MMSW with commercial and non-commercial sexual partners, as discrepancies have been reported in the use of condom according to the type of sexual partner, ^16^^,^^18^^,^^44^^–^^46^ and the sexual role performed during the sexual act. ^42^ This paper aims to describe epidemiological and contextual aspects of HIV-STI transmission and contribute with recommendations for future educational interventions for this highly vulnerable group. Findings will contribute to promote the use of condoms as part of the combination on prevention of HIV infection to enhance the effectiveness of PrEP and PEP to decrease the number of new HIV and STI infections.

## Methods

### Study design and recruitment

This qualitative research was carried outed between May 2013 to August 2015. Convenience sampling method was used to recruit men aged 18 and over, who were non-British born, lived in the UK for at least one year, and who had worked or were still working as sex workers. The main recruitment sites were sexual health clinics and projects in London that provide health services and social services to different vulnerable groups, and among them sex workers. Health professionals and health workers took part in the recruitment by providing potential participants (who meet the study criteria) with the participant information sheet (PIS) and flyers. Those who were interested in take part of the study contacted directly the researcher to receive additional information and organise the interview.

### Data collection and analysis

An interview guide was prepared using pertinent literature and first-hand information obtained from key informants (researchers and health professionals) who provide health and social services to sex workers. This guide was piloted on three MMSW, however the results were not included in the final analysis. The interview guide included questions about their experience as male escorts, in that manner they were asked about type of sexual services offered, characteristics of their customers, how they elaborate their escort adverts, use of alcohol and recreational drugs with clients, recall events of condomless sex with clients and within non-commercial sexual partners in the last 12 months, type of non-commercial sexual partners, how they define these non-commercial sexual partners ( Underlying data). ^47^

Participants could select the place for the interview. They were offered counselling room in St. Marys hospital and one-to-one meeting rooms in the City, University of London. These places were approved by the Ethics Committee and were accessible to participants to secure their privacy. Literal transcription of the voice recordings were printed and revised by each participant to confirm their accuracy before the analysis. In this manner, the risk of misinterpretation due to cultural backgrounds and misunderstanding was minimised. Thematic analysis was conducted using ATLAS.ti version 8.0. ^48^ Coding rules and a clear process to identify and define themes emerging from the data were established to avoid ambiguity or inconsistency. The coding performed by the researcher was revised and corroborated by the supervisors. When it was a conflict about coding between the researcher and supervisors, this was resolved and applied to the data. For this investigation, ‘condomless sex’ was defined as any penetrative or receptive sexual intercourse (oral, vaginal, or anal) without using condoms. Conversely, ‘safer sex’ was specified as the use of condoms for the aforementioned practices. The emergent themes were interpreted using the theory of planned behavior (TPB) ^49^ framework. This theory is based on the significance of attitudes, norms, and perceived control to explicate different forms of risky behaviours, ^49^ and to plan health interventions. ^50^ In this manner, the analysis was focused on 1) attitudes towards the use of condoms: a result of personal beliefs about condoms; 2) subjective norms derived from participants’ perception of what others think about condoms (normative beliefs) and their motivation to comply with norms, and 3) perceived behavior control: participant’s beliefs about the degree of control they have over the use of condoms during the intercourse.

### Ethical considerations and consent

This study was revised and approved by the Ethics Committee of City, University of London (18 April 2013, Ref: PhD/12-13/18), by the NRES London Central Committee (9 October 2013, Ref:13/LO/1306, IRAS ID: 132947), and by the Research Committee of St. Mary’s Hospital (15 January 2014, Ref:13SM1864). Written informed consent was obtained from all individual participants included in the study. This form was revised and approved by the ethics committee.

## Results

### Disclaimer

Due to the explicit nature of the interviews some quotes have been edited for clarity.

### Main characteristics of the participants

In this study a total of 25 MMSW took part in this study. This sample was almost evenly represented by men from Latin-America and Europe. The socio-demographic characteristics of participants as well as their patterns of migration, and entrance into sex work suggest their diversity. Almost all of participants (22/25) had been diagnosed with one or more STIs including HIV ( Table 1). The whole group was operating as independent internet-based escorts, providing sexual services to men and women. The latter in the context of sexual services for ‘couples’ (man and woman). The analysis shows two dominant themes and distinct subthemes:

**Table 1.** The socio-demographic, immigration status and sexual health characteristics of participants.

Home-country	Total (n = 25)
Brazil	10 (40%)
Colombia	02 (8%)
Bulgaria	02 (8%)
Spain	06 (24%)
Italy	02 (8%)
Portugal	01 (4%)
Latvia	01 (4%)
Nigeria	01 (4%)
**Age** (mean [range], years)	33 (24-44)
**Patterns of immigration to the UK**	
Direct migration	14 (56 %)
Multi-stage migration	11 (44%)
Age at emigration (mean [range], years)	24 (13-40)
Years living in the UK (median [range], years)	06 (1-23)
**Entrance into sex work**	
Age (median [range], years)	27 (14-41)
Years in sex work (mean [range], years)	06 (1-16)
**Level of education achieved**	
Primary	02 (8%)
Secondary	10 (40%)
Further/higher education	13 (52%)
**Sexual orientation reported**	
Gay	19 (76%)
Bisexual	06 (24%)
**Currently using recreational drugs at work**	
Yes	15 (60%)
No	10 (40%)
**Currently using recreational drugs in personal life**	
Yes	18 (72%)
No	07 (28%)
**STI reported**	22/25 (88%)
Syphilis	12 (48%)
Gonorrhea	15 (60%)
Chlamydia	16 (64%)
Herpes	02 (8%)
Genital warts	03 (12%)
HIV	03 (12%)

**A.** Condom use with clients

This theme explains the perspective of the use of condoms within the context of sex work and experiences of condomless sex that contains three sub-themes:

1. Attitude towards the use of condoms and policy on condom use with clients

It describes the attitude and perceived norm about the use of condoms with clients. The statements indicate that the entire group of participants were aware of the HIV and other STIs, claimed to be risk averse, and more importantly, stated a consistent use of condoms as a perceived norm because of their work. In line with this, many of them have made explicit their rejection to condomless sex in their online adverts:

“Always, always condom, nothing without a condom, never, ever, ever. They can pay me any money, there are some offers, and some people asked me - do you do [condomless sex]? For that reason, in my profile I wrote no [condomless sex], don’t even ask me”

Consistently, the majority of participants expressed a favourable attitude towards safer sex. For some, using condoms with clients was a way to differentiate sexual services from having sex in their personal life. Others thought that condoms were useful devices to avoid poor hygiene, odour, and some bodily features of clients that they find unattractive or dislike (e.g., overweight, excessive corporal hair). Most of the participants said that they especially demand the use of condoms to provide services such as vaginal intercourse, ‘full-service’ for men (usually include anal sex) and performing “passive” sexual role service (receptive anal sex):

“I offer a full service, a complete service but I try to be specific because people always ask you if you do without condom, So I always say condom, I won’t give you my phone, I am a very discrete person and I only do outcalls.”

By contrast, other participants admitted an unfavourable attitude towards condoms. While the most mentioned concerns of losing clients that reject condoms, few did not like their use as it reduced their sexual arousal, especially with erection which caused difficulties in their sexual performance:

“I know that I won’t have many clients if I insist on having oral sex with a condom, so I prepare to take that risk that is the only one that I prepare to take the chances.”

*“I don’t enjoy at all when I use a condom for [oral sex] because it is like you are sucking a rubber and I just get soft* [lose the erection] *when I wear a condom for a [oral sex], once again it is because of it something like squeeze it is a bit weird.”*

The discrepancy between these two different perspectives suggests that risk awareness and intention of using condoms do not guarantee safer sex.

In addition, the analysis of the narratives showed that condomless sex with clients occurred in two different scenarios. In the first scenario participants perceived control of the situation and made a risk-taking decision to dismiss the use of condoms, and in the second, they lost control of the situation that ended in a condomless sex event. These are described under the following sub-themes:

2. Risk-taking decision to accept condomless sex

This sub-theme explains the decision-making process that participants applied to dismiss the use of condoms with clients. They used two main processes a risk assessment of the clients, and performing sexual practices that participants catalogued as *‘low-risk’* in contracting HIV and other STIs. The participants assessed the risks of the client based on their physical appearance, followed by a subtle physical examination to identify sores, warts or blisters on genitals or rectum, or presence of penile discharge. Through this practice participants accepted condomless sex with customers that were labelled as *‘healthy’* and *‘clean’* (e.g., absence of warts):

“I [had sex with] him all without condom, but I was not a risk as I could tell that I was probably the second or third person who he has sex with. I think I could tell about everything I do not think he has anything because he probably has very little sexual life.”

The participants also considered some social and demographic characteristics to rank clients as *‘low risk’* or *‘high risk’.* In this instance, participants favoured clients who were *‘married men’* for condomless sex as they were perceived as *‘straight’* men who *‘only have sex with their wives.’* In the same way, participants considered their *‘regular’* clients with whom they had established a long-term and trustworthy relationship, as ‘low risk’:

“For example, yesterday I had a man from Barbados who looked very healthy, but I know he is from a high-risk region for these diseases. The guy was very clean, he was very nice”

“There are a couple of people that I don’t use a condom because I know them for quite long time. I know it is not a good policy. I know I should use a condom with everybody and that’s it, but there are few people that I do that.”

A second procedure to accept condomless sex with clients was the selection of sexual practices that participants considered as *‘low-risk.’* By far, condomless oral intercourse (COI) was the most frequent practice. Some participants mentioned that as additional strategies of protection they reduce the time for COI and avoid contact with the client’s semen. Although, condomless insertive oral intercourse (CIOI) and condomless receptive oral intercourse (CROI) were equally reported by participants, some assigned different levels of risk to each:

“I know it is less risky when I suck him than when he sucks me or to kiss him. But it is not for everybody, depend on of the situation”.

Another recurrent risk reduction practice mentioned was performing condomless anal sex as the active sexual role (penetrative anal intercourse) instead of a passive role (insertive anal intercourse), as it was perceived as less risky:

*“Part of me think I am mainly top, I normally do not get people to [have sex with] me, I [have sex with] them, I am mainly top, and that is a very low risk,* [censored] *! I am not at risk because it is the very little rate to catch something if I am mainly top.”*

“I think, I am in this scene, I am earning money, but I am also scared because this is very risky, but I always pray to God please nothing bad happen.”

3. Contextual factors determining condomless anal sex

This sub-theme describes the role of four main factors that made participants to *‘lose control’* or to be under pressure to perform condomless anal intercourse. One of the most recurrent conditions was the use of recreational drugs that provoked a strong sexual arousal among participants. Many of these events occurred when they were providing *‘overnight’ or ‘chemsex’* services:

*“With crystal meth your brain is still more there, but with mephedrone you do not even think straight so much, you are so* [aroused] *that you do not even think, you only want sex, if I take mephedrone I know I am not going to use condoms”*

A second recurrent and independent condition was feeling strong attraction to a client:

“Actually, I wasn’t on drugs, this time I wasn’t on drugs, I came to see this guy in the Ritz Hotel, and he was an Arabic, he was about my age, and he was so gorgeous! Sexy, he was like my God! I just wanted to eat him alive, he was so sexy and then, you know what actually I did it without condom”

A third factor driving condomless anal sex was the financial reward offered by clients, which was mostly reported by participants who were in precarious conditions. In these situations, the participants felt that they could not reject the offer:

“I had a client once, the same client three times because that client, he pays very well, much more that what I asked”.

“I am at risk if you ask me how I feel about it, not very safe, not very clever. But I gamble for the best, I need the cash, I need the cash for food, I need the cash for transport, and I need to get out of this hole because I smile when I meet new people and everything, but when I am alone is not easy.”

“Once I was really bad about money and a client called me and he wanted to take drugs also if you don’t take drugs, you can last all that you need or you can even cope with the client.”

The fourth condition describes scenarios in which participants were overpowered by clients who removed or broke condoms or took advantage of the dynamic during the sex session to perform condomless anal intercourse. This condition was usually reported by the participants who offer a ‘passive’ sexual role as part of their sexual services:

“I was having sex with a guy who was doing as active, and you know suddenly I saw him with the condom on his hand and I asking him, ‘Were you [having sex with] me without a condom?!”

In few cases, participants reported that these events occurred in a context of physical and verbal violence perpetrated by clients, or within a context of drugs use:

“We were taking cocaine and drinking, I drank so much that day and I passed out […] few hours later, I woke up and the reason that I woke up was because something was painful, ok? And the painful thing was that he was [having sex with] me on my sleep, he was [having sex with] me on my sleep and without condom.”

B. Condom use in private life

This theme describes experiences of unpaid or non-commercial condomless sex, which was defined as sexual acts without the use of condoms that were performed without any intention of material or economic reward. In general, many participants declared a more inconsistent use of condom with non-commercial sexual partners than with clients:

“Then it happened again, but he wasn’t a client he was a person that I met, a casual partner and again it was three months of waiting for the test and I was - Oh my God, I shouldn’t do it again!”

“I haven’t been in risk. My sexual practices are very low risk in the context of work, and the only person with I have been in high risk is with my ex-partner. During the time when we knew that he got infected we used protection until he completed the treatment”

This theme contains two sub-themes to differentiate condomless sex practice with formal sexual partners from casual sexual partners. Most of the participants reported having casual partners along with a formal partner in the past year.

The sub-themes are described below:

1. Condomless anal sex with formal partner

The category of formal sexual partner was used by participants to describe people with whom they had a romantic, stable/regular or committed relationship. Almost half of the group reported to have male formal sexual partners. Some mentioned that these partners were also working as escorts, even few worked together. Most importantly, majority reported an irregular or complete lack of condom use with these partners. They decided not to use condoms when their partners agreed to just have sex with them, and/or knew both were HIV negative:

“When I am dating someone if we both checked [got tested for HIV] and we both are fine, we do not use condom, like my ex that we split up two months ago, we were together for a year as we never use condoms, but I knew he only was sleeping with me”

For these participants condomless anal sex represented pleasure, intimacy, and commitment with their partners:

“Have sex without condom with my ex-partner wasn’t good idea, even if that gives me more pleasure and it gives me more intimacy because sex between us hasn’t been the most important part of our relationship”

Coherently, few participants said that they *‘always’* used condoms with their formal partners because they knew that one of them was HIV positive (serodiscordant couples):

“He found out that he was HIV positive and then at that time I got syphilis from him and at that time I wasn’t working as an escort I was working as a cleaner and I didn’t get the HIV, so I got treated for syphilis, he got treated as well and from then we started to have sex with condom.”

2. Condomless anal sex with casual partners

Casual sexual partner was defined as people with whom participants engaged in sexual intercourse without a sense of commitment or emotional attachment. They mainly met casual partners using dating mobile applications, websites or in places such as clubs, saunas, or clubs. These participants decided not to use condom with these partners to satisfy their pleasure and personal enjoyment. Some admitted that they perceived the use of condom was optional:

“ *We are humans and sex is the most animal part of us, you know, we are animals completely, so you cannot always control it, you have to accept it, if you always are having sex […] that is why you do without condom and see what happen.”*

“If someone carry a condom, then we do it with condom, or we just leave the condom around and try to see how it goes.”

However, it is important to mention that many participants also acknowledged the role of recreational drugs and alcohol consumption as well as feeling sexually attracted to casual sexual partners in the practice of condomless anal intercourse:

“When you are in drugs the only thing that you want is to have sex, well it depends, in my case I only wanted to have sex, for free, sex with people that if I could be rational, I wouldn’t like to have sex with, and exposing yourself to catch anything.”

“The very last time was about 6 months ago and that was with a neighbour, a very, very hot Spanish guy who came around and we had some fun and when he started [having sex with] me without condom”

## Discussion

This study has used the lenses of the theory of planned behaviour to understand the condomless sex behaviour of 25 MMSW with commercial and non-commercial sexual partners. Participants were aware of HIV-STI and self-perceived at risk because of their sex work. Unlike previous research, ^51^ they claim being risk adverse and consistently declared the norm of using condoms in their escort adverts as a normative expectation of others. However, despite the statements showing a positive attitude towards the use of condoms as a perceived norm, and consequently their intention of using them, participants revealed that condomless sex was a frequent practice. ^52^^–^^54^ They explained that do not like condoms because reducing their sexual arousal, and consequently their sexual response (erection), which is an issue providing sexual services. This aspect, not so often acknowledged, highlights the significance of the sexual performance in this type of work, ^55^ that can reduce the behavioural intention that predict the likelihood of using condoms. The narrative of recent events of condomless sex with clients unveiled a perceived behavioural control among participants every time that they had to make a decision of dismissing the use of condoms. They described this risk-taking decision as an intuitive process of risk assessment of the client based on their knowledge, beliefs and experiences about the transmission of HIV-STI. In this manner, they searched for physical evidence of these infections in the clients’ bodies and evaluated their social and demographic characteristics to which they attributed risk. Further, to lessen the risk of transmission they decided to perform sexual practices considered as ‘low risk’. Notwithstanding, these practices that were described as habits or part of their work routine corroborates self-protective behaviour among participants, the knowledge that they used for risk management demonstrated the persistence of inaccurate information about HIV and other STIs. ^17^^,^^56^^–^^59^ In addition, narratives indicate that condomless sex with clients also occurred when participants consciously lose perceived control and dismiss the initial intention of safe sex and decided to perform condomless anal sex. Among the contextual factors driving this behaviour, one of the most frequent reported was the use of recreational drugs with clients. ^60^^–^^62^ Drug use has been described as a social aspect of this type of work ^63^ which makes difficult to avoid with clients who are regular drug users, ^61^ and they are also used to improve sexual performance. ^64^ About the latter, recreational drugs initiate sexual interaction, ^52^^,^^65^^–^^67^ causing sexual arousal that facilitates sexual acts. ^68^^,^^69^ Another important factor was feeling sexually attracted to the client, which made participants to indulge in personal sexual pleasure. ^70^^,^^71^ Less reported among our participants were the precarious situation that made them to accept financial reward in exchange for condomless sex ^72^^–^^75^ as well as physical domination and verbal violence perpetrated by clients. ^72^^,^^75^ All these factors contextualise condomless experience in scenarios of vulnerability for MMSW. ^76^ These findings challenge claims that recreational drugs are not problematic among male escorts, ^77^^–^^79^ that they work in safer environments, obtain higher earnings, and can control work conditions. ^78^^,^^80^^,^^81^

Within the context of private life, this study found differences in the background factors for condomless sex according to the type of sexual partner. As such, the use of condoms was not considered with formal partners due to normative beliefs attributed to emotional attachment linked to this type of relationship. Condoms were also dismissed when participants and their partners were both HIV negative and agreed to keep condomless sex strictly among themselves as a norm. Yet some of these participants informed episodes of STI associated to their formal partners. Conversely, the use of condoms was agreed as a norm when they were a HIV-serodiscordant couple ^55^^,^^82^ Having casual sexual partners was a recurrent behaviour even among those who reported formal partners. Condomless sex with this type of sexual partners was frequent, particularly among participants who declared this practice with clients. In this way, this study corroborates that condomless sex with casual partners can be a predictor of condomless sex with clients. ^70^ Besides, participants connected condomless sex to the use of recreational drugs, strong attraction and sexual arousal. This is a significant contextual factor as recreational drugs affect the perception and response to risk, leading the behaviour to high-risk sexual practices, ^83^ that increase the likelihood of contracting HIV and other STIs. ^84^^,^^85^ in this manner the finding support the perspective that the type of sexual partner chosen in the MSW’s personal life can also be a risk factor. ^52^^,^^65^

In terms of beliefs about the likely consequences of their unsafe sex behaviour, having anal condomless sex was the main reason that motivated participants to visit sexual health clinics to have screening tests for HIV and other STIs. They were more concerned about HIV than other STI. Almost the entire group had requested PEP ^86^^,^^87^ more than twice in the last 12 months. Few admitted that did not complete the treatment due to the adverse effects. This finding raise concerns of possible seroconversions when considering the poor medication adherence. ^88^ Based on the findings described above, it is possible to suggest that the use of recreational drugs and feeling attracted to a sexual partner were relevant contextual factors that intersect the private sexual life and sex work experiences of our participants. In summary, the findings of this study corroborated that the applicability of the theory of planned behaviour to assess risky sexual behaviour among vulnerable groups such as MMSW. ^89^^,^^90^ Still, further investigation is needed to confirm potential moderating effects of contextual factors that were described here and can aid the generation of suitable measures of the persistent condomless sex behaviour among MMSW.

### Strengths and limitations

Interpretation of the findings and the evaluation of their significance should be made considering the limitations of this study. For instance, the qualitative study design prevents the generalization of the findings. Also, limiting the recruitment of participants to sexual health clinics and health projects in London due to the recommendation of the research ethics committees, restrict the results only to the perspective and experiences of migrants who attend these services. Nonetheless, even with these limitations, this study is one of the few on male migrant sex workers in the UK that captures their experiences of high-risk sexual behaviour in detail. In addition, the heterogeneity of the sample provided a rich qualitative data on MMSW’s risky sexual behaviour with commercial and non-commercial sexual partners. Furthermore, this study provides in-depth socio-behavioural insights for designing more effective and tailored interventions for MMSW self-identified as homosexuals and bisexuals.

## Conclusions

Despite that participant declared a positive attitude, normative beliefs, and intentions of using condoms with commercial sexual partners, this study found that condomless sex was a recurrent practice. Beliefs about condoms causing issues in their sexual response that make difficult to perform their sexual services was reported. In addition, condomless sex with clients occurred in scenarios of perceived control in which participants made a risk-taking decision that intuitively triggered a set of risk reduction practices that may not work effectively as they were based upon myths and misinformation about the HIV-STI transmission. However, condomless sex with clients also occurred in a context of perceived loss of control when they used recreational drug, felt attracted to a client, were experiencing precarious conditions, physical domination and verbal violence perpetrated by clients. Condomless sex with non-commercial sexual partners was also a common practice, but the attitude towards the use of condoms was regulated by meanings and emotions that participants attributed to formal sexual partners and casual sexual partners. Contextual factors that determined the dismissal of condoms with formal partners were related to HIV status and couple’s agreement to maintain this practice among them. Conversely, contextual factors linked to condomless sex with casual partners were the use of recreational drugs and feeling attracted to the casual partner. Then, it is possible to suggest that the use of recreational drugs and feeling attracted to a sexual partner were relevant contextual factors that intersected private sexual life and the sex work experiences of our participants. The findings highlight the potential of using the TPB to better understand condomless sex practice among MMSW with different types of sexual partners. Further research is needed to provide deep insights about perceived risk, meanings attributed to these infections, and STI-HIV prevention measures that help to tailor appropriate interventions.

## Recommendations

To comprehensively address the transmission of HIV-STI among MSW is important to acknowledge the significance of groups at greater risk as migrants in the UK. National policies that introduce approaches to better engage migrants in the use of health services are needed not just from a public health perspective, but also as principle for social justice and human rights. Likewise, appropriate surveillance, funding, and a multilevel intervention to tackle the variety of MMSW and their needs can successfully improve the number of MMSW using prevention programmes. Designing suitable programmes using scientific evidence, but also MMSW experiences to reinforce educational interventions that correct misinformation about the transmissibility of HIV and other STI can make these interventions more relatable. Equally important is strengthen the risk-reduction counselling for those requesting PrEP and PEP to promote the use of condoms, condom negotiation, skills of self-control and the compliance of these treatments to secure their effectiveness. It is essential to train healthcare professionals to identify vulnerable sub-groups among MMSW such as those using recreational drugs to offer them a referral to programmes of harm reduction in substance use and mental health services. In the same manner, the identification of MMSW whose partners are also sex workers, have a HIV serodiscordant partner, tend to have condomless sex with casual sexual partners, and are experiencing difficult-living or working conditions as they will need specific support.

## Data availability

### Underlying data

Repository: Perceived risk and condomless sex practice with commercial and non-commercial sexual partners of male migrant sex workers in London, UK https://figshare.com/s/cbf4a21a9d93d63472c8. ^47^

This project contains the following underlying data:

• File docx. This file contains the blank interview questionnaire.

Data are available under the terms of the Creative Commons Zero “No rights reserved” data waiver (CC0 4.0 Public domain dedication).
