# Supplementary material for: Perceived risk and condomless sex practice with commercial and non-commercial sexual partners of male migrant sex workers in London, UK
Source: F1000Res. 2023 Oct 19;10:1033. Originally published 2021 Oct 11. [Version 2] doi: 10.12688/f1000research.73248.2 (PMC10622860; doi:10.12688/f1000research.73248.2)
Supplement: Peer review history

# Reviewer report: Reviewer response for version 2

**DOI:** 10.5256/f1000research.156882.r217083

**Published:** 2023-11-02

**Authors:** Carlos F. Caceres

**Affiliations:** 1 School of Public Health and Administration, Center for Interdisciplinary Studies in Sexuality, AIDS and Society, Universidad Peruana Cayetano Heredia, Lima, Peru

The manuscript has been revised by the author. Most of this reviewer's recommendations have been followed reasonably well; others seem to depend not on the author but on the journal (e.g. about quotes, it would have been useful to have some information on the specific participants that could give an idea of diversity of voices [codes] or on sociodemographics [age, nationality]). It would be helpful to clarify that data were collected at a time when PEP and PrEP were not fully available, as their existence changes the interpretation of risk-taking significantly.

 Importantly, the text still needs professional  copy-editing.

Is the work clearly and accurately presented and does it cite the current literature?

Partly

If applicable, is the statistical analysis and its interpretation appropriate?

Not applicable

Are all the source data underlying the results available to ensure full reproducibility?

No

Is the study design appropriate and is the work technically sound?

Yes

Are the conclusions drawn adequately supported by the results?

Yes

Are sufficient details of methods and analysis provided to allow replication by others?

Yes

Reviewer Expertise:

HIV and sexual health

I confirm that I have read this submission and believe that I have an appropriate level of expertise to confirm that it is of an acceptable scientific standard.

# Reviewer report: Reviewer response for version 2

**DOI:** 10.5256/f1000research.156882.r217082

**Published:** 2023-10-24

**Authors:** Prof Victor Minichiello

**Affiliations:** 1 Australian Research Centre in Sex, Health and Society, La Trobe University, Melbourne, VIC, Australia

I am happy with the revisions.

Is the work clearly and accurately presented and does it cite the current literature?

Partly

If applicable, is the statistical analysis and its interpretation appropriate?

Not applicable

Are all the source data underlying the results available to ensure full reproducibility?

Yes

Is the study design appropriate and is the work technically sound?

Partly

Are the conclusions drawn adequately supported by the results?

Yes

Are sufficient details of methods and analysis provided to allow replication by others?

Partly

Reviewer Expertise:

sex work, qualitative data analysis

I confirm that I have read this submission and believe that I have an appropriate level of expertise to confirm that it is of an acceptable scientific standard.

# Response

**Published:** 2023-10-24

**Authors:** Elisa Ruiz

**Affiliations:** University College London, UK

Dear Prof Minichiello,

 Many thanks for revising and approving this version. I really appreciate your valuable input.

 Best regards,

 Elisia

# Reviewer report: Reviewer response for version 1

**DOI:** 10.5256/f1000research.76889.r169988

**Published:** 2023-05-15

**Authors:** Carlos F. Caceres

**Affiliations:** 1 School of Public Health and Administration, Center for Interdisciplinary Studies in Sexuality, AIDS and Society, Universidad Peruana Cayetano Heredia, Lima, Peru

This is an interesting report about a relevant topic for the UK and worldwide. To make it more suitable for indexing, however, a few issues should be resolved:

1. The most visible one is the slang of the quotations (of migrant male sex workers who don't speak English as a first language). Probably there are guidelines as to how they should be edited.
2. Likewise, there are some issues with the English language writing of the main text that limit its clarity and should be resolved.
3. The MMSW quotations should ideally include a code assigned to each participant, as well as their nationality. That would help interpreting the findings.
4. The introduction fails to contextualize the discussions in a framework of combination HIV prevention. Treatment as prevention (and U=U, that is viral suppression as risk-reducing) is never mentioned, PEP is mentioned once, and PrEP is presumably mentioned ("pre-PEP") in the recommendations only. It is unclear whether people who are having condomless sex are really out of any form of protection.
5. In that sense, the dates of implementation of this study should be specified, for better interpretation of the context.
6. In A.1, when describing unfavourable attitudes towards condoms: (a) the authors inappropriately use condom use in oral sex to illustrate the dilemmas of condom use, while it is not that relevant given its very low risk; (b) authors conflate MSW's fear of others rejecting condoms, with their own predisposition against condoms - those should be presented separately, although certainly with regard to anal, not oral sex.
7. The theory of planned behaviour should be used at least in the discussion.
8. The discussion should address other options of combination HIV prevention. Either at the introduction or at the discussion, current access to treatment, PEP and PrEP should be discussed as alternatives to condom use.

Is the work clearly and accurately presented and does it cite the current literature?

Partly

If applicable, is the statistical analysis and its interpretation appropriate?

Not applicable

Are all the source data underlying the results available to ensure full reproducibility?

No

Is the study design appropriate and is the work technically sound?

Yes

Are the conclusions drawn adequately supported by the results?

Yes

Are sufficient details of methods and analysis provided to allow replication by others?

Yes

Reviewer Expertise:

HIV and sexual health

I confirm that I have read this submission and believe that I have an appropriate level of expertise to confirm that it is of an acceptable scientific standard, however I have significant reservations, as outlined above.

# Response

**Published:** 2023-09-30

**Authors:** Elisa Ruiz

**Affiliations:** University College London, UK

Dear Carlos, 

 Thanks very much for your comments and suggestions that helped me to improve the paper. Please see my answers below:

 Reviewer comment: The most visible one is the slang of the quotations (of migrant male sex workers who don't speak English as a first language). Probably there are guidelines as to how they should be edited.

*Author answer: the quotes have been edited by the editorial team of this journal according to their internal policies. In this manner, some words have been removed (as state the disclaimer section (in Results)*

 Reviewer comment: Likewise, there are some issues with the English language writing of the main text that limit its clarity and should be resolved.

*Author answer: The text has been amended to improve their clarity.*

 Reviewer comment: The MMSW quotations should ideally include a code assigned to each participant, as well as their nationality. That would help interpreting the findings.

*Author answer: variables as age, pseudonym and country of birth were part of the initial version of this manuscript, but they were removed by the editorial team of this journal. I will be happy to restore this information if they agree to this.*

 Reviewer comment: The introduction fails to contextualize the discussions in a framework of combination HIV prevention. Treatment as prevention (and U=U, that is viral suppression as risk-reducing) is never mentioned, PEP is mentioned once, and PrEP is presumably mentioned ("pre-PEP") in the recommendations only. It is unclear whether people who are having condomless sex are really out of any form of protection.

*Author answer: I really appreciate this suggestion that open an interesting scope for further research. However, it is important to mention that the data collection for this study took place between 2013-2015, while PrEp and PEP initiatives were increasingly available  since 2016. Participants who had access to them was mostly because were part of trials.*

 Reviewer comment: In that sense, the dates of implementation of this study should be specified, for better interpretation of the context.

*Author answer: This info has been updated in abstract and methods section*

 Reviewer comment: In A.1, when describing unfavourable attitudes towards condoms: (a) the authors inappropriately use condom use in oral sex to illustrate the dilemmas of condom use, while it is not that relevant given its very low risk; (b) authors conflate MSW's fear of others rejecting condoms, with their own predisposition against condoms - those should be presented separately, although certainly with regard to anal, not oral sex.

*Author answer: I understand this point, however, condomless sex was examined as an approach for HIV-STI prevention.*

 Reviewer comment: The theory of planned behaviour should be used at least in the discussion.

*Author answer: Discussion section has been amended to satisfy this suggestion.*

 Reviewer comment: The discussion should address other options of combination HIV prevention. Either at the introduction or at the discussion, current access to treatment, PEP and PrEP should be discussed as alternatives to condom use.

*Author answer: the introduction section has been amended to satisfy this recommendation.*

# Reviewer report: Reviewer response for version 1

**DOI:** 10.5256/f1000research.76889.r96637

**Published:** 2021-10-14

**Authors:** Prof Victor Minichiello

**Affiliations:** 1 Australian Research Centre in Sex, Health and Society, La Trobe University, Melbourne, VIC, Australia

Understanding how male sex workers make decisions about condom usage with clients and in their personal sexual lives is an important question that this study addresses. Recent studies have explored this topic in some detail. This study aims to contribute by interviewing migrant male escorts in London and offering explanations that delve into why sex with a condom occurs or not in their encounters with clients.

 This is an interesting paper, but there are several gaps:

 First, the literature review is not complete. I suggest the author read the recent work by John Scott and his colleagues on male sex work: see *The Routledge Handbook of Male Sex Work, Culture and Society* published by Routledge this year to better grasp the literature on this topic.

 Second, could the author expand on new insights for public health campaigns concerning PrEP and HIV prevention that emerge from this study, and better argue how the results of this study further advance knowledge on the topic of condom use and safe sex among male escorts and particularly vulnerable groups like migrant sex workers?

 Third, some methodological issues require addressing. What topics were included on the interview guide? How were the interviews conducted? Can more detail be given about how the data themes were developed and what sort of qualitative data analysis was used? 

 Finally, what new insights for public health campaigns emerge from this study?

Is the work clearly and accurately presented and does it cite the current literature?

Partly

If applicable, is the statistical analysis and its interpretation appropriate?

Not applicable

Are all the source data underlying the results available to ensure full reproducibility?

Yes

Is the study design appropriate and is the work technically sound?

Partly

Are the conclusions drawn adequately supported by the results?

Yes

Are sufficient details of methods and analysis provided to allow replication by others?

Partly

Reviewer Expertise:

sex work, qualitative data analysis

I confirm that I have read this submission and believe that I have an appropriate level of expertise to confirm that it is of an acceptable scientific standard, however I have significant reservations, as outlined above.

# Response

**Published:** 2023-09-30

**Authors:** Elisa Ruiz

**Affiliations:** University College London, UK

Dear Victor,

 I am very grateful for your comments and suggestions to improve this paper.  Please see my answers below:

 Reviewer comment: "The literature review is not complete. I suggest the author read the recent work by John Scott and his colleagues on male sex work: see *The Routledge Handbook of Male Sex Work, Culture and Society* published by Routledge this year to better grasp the literature on this topic"

*-Author answer: I have updated the literature including also some significant information from the source suggested by this reviewer.*

 Reviewer comment: "Second, could the author expand on new insights for public health campaigns concerning PrEP and HIV prevention that emerge from this study, and better argue how the results of this study further advance knowledge on the topic of condom use and safe sex among male escorts and particularly vulnerable groups like migrant sex workers?"

*-Author answer: the recommendation section has been updated to highlight the promotion of condom use, as important element of the combine prevention of HIV.*

 Reviewer comment: "Third, some methodological issues require addressing. What topics were included on the interview guide? How were the interviews conducted? Can more detail be given about how the data themes were developed and what sort of qualitative data analysis was used?" 

 - *Author answer: Additional information required by the reviewer has been added to the methods section.*

 Reviewer comment: "Finally, what new insights for public health campaigns emerge from this study?"

 - *Author answer: Recommendation section has been updated to address this suggestion,*
